# Fasziokutane Brückenlappenplastik zur Defektdeckung im Komplikationsfall am Unterschenkel nach Kompartmentsyndrom: Eine „fast“ vergessene, sichere Lappenplastik

**DOI:** 10.1007/s00113-024-01481-7

**Published:** 2024-09-20

**Authors:** Anton Borger, Tobias Karge, Rita Babeluk, Lukas Zak, Lorenz Semmler, Stefan Hajdu, Christine Radtke

**Affiliations:** 1 Universitätsklinik für Plastische Rekonstruktive und Ästhetische Chirurgie, Medizinische Universität Wien/Allgemeines Krankenhaus Wien, Spitalgasse 23, 1090 Wien, Österreich; 2 Austrian Cluster for Tissue Regeneration, Wien, Österreich; 3 Klinische Abteilung für Unfallchirurgie, Universitätsklinik für Orthopädie und Unfallchirurgie, Medizinische Universität/Allgemeines Krankenhaus Wien, Wien, Österreich

**Keywords:** Kompartmentsyndrom, Lappenplastik, Orthoplastik, Weichteildefekt, Compartment syndrome, Surgical flaps, Soft tissue injuries, Orthoplastics

## Abstract

Wir berichten hier von einem komplizierten Fall eines Weichteildefektes mit einer herausfordernden Weichteildeckung am rechten Unterschenkel. Ein 29-jähriger Mann entwickelte nach einer Unterschenkelfraktur und Versorgung mit einem Tibianagel ein Kompartmentsyndrom aufgrund einer massiven Nachblutung mit einer Läsion des N. peronaeus communis sowie Muskelnekrosen im Bereich der Fibularisloge. Die initiale Deckung mit Spalthaut zeigte keine Heilungstendenz, sodass der Patient mit einem Weichteildefekt von ca. 25 × 10 cm am lateralen Unterschenkel mit frei liegender Tibia über 15 cm Länge in unser Krankenhaus übernommen wurde. Es erfolgte primär der Deckungsversuch mit einer Spalthauttransplantation nach sekundärer Granulation. Aufgrund einer vorgeschädigten Gefäßversorgung zeigte sich allerdings über 8 Monate eine frustrane Wundheilung. Zudem fielen im Röntgen eine Pseudarthrose und ein daraus resultierender Schraubenbruch der beiden distalen Verriegelungsschrauben auf. Hierbei wurde die Indikation für einen Revisionseingriff zur Frakturversorgung und zum Implantatwechsel gestellt. Im gleichen Eingriff wurde der Verschluss der kutanen Restdefekte durchgeführt. Bei einem bisher komplikationsbehafteten Verlauf und der schwierigen lokalen Durchblutungssituation war die Auswahl der Rekonstruktionsverfahren begrenzt. Es wurde in einem interdisziplinären Vorgehen eine Brückenlappenplastik vom medialen Unterschenkel durchgeführt. Der Hebedefekt wurde mit Spalthaut gedeckt. Die Wunde konnte auf diese Weise nach einem Jahr schließlich suffizient gedeckt werden.

## Abstract (en)

This article reports on a complicated case of a soft tissue defect with challenging soft tissue coverage on the lower leg. After a lower leg fracture and treatment with a tibial nail, a 29-year-old man developed compartment syndrome due to massive secondary bleeding with a lesion of the common peroneal nerve and muscle necrosis around the fibular muscles. The initial coverage with split skin showed no tendency to heal, so the patient was admitted to this hospital with a soft tissue defect of approximately 25 cm × 10 cm on the lateral lower leg with an exposed tibia over a length of 15 cm. The primary attempt was coverage with a split-thickness skin graft after secondary granulation; however, due to the previously damaged vascular supply, the wound demonstrated a delayed incomplete healing over 8 months. In addition, X‑ray imaging revealed a nonunion and a resulting screw fracture of the two distal locking screws. The indications for revision surgery to treat the fracture and change the implant were fulfilled. In the same procedure, the residual cutaneous defects were closed. Given the previously complication-prone course and a difficult local blood flow situation, the choice of reconstruction procedures was limited. A bridge flap of the medial lower leg was performed in an interdisciplinary approach. The lifting defect was covered with split-thickness skin. In this way, the wound was finally adequately covered after 1 year.

## Hintergrund

Ein zentrales Konzept der rekonstruktiven Chirurgie stellte die „rekonstruktive Leiter“ dar [1]. Mittlerweile wurde das Konzept überarbeitet, und weitere Überlegungen sind miteingeflossen [2–4]. Es gilt grundsätzlich einen Defektverschluss mit maximalem Vorteil für den Patienten anzustreben. Dabei werden Verfahren wie eine sekundäre Heilung, Hauttransplantationen sowie lokale und freie Lappenplastiken eingesetzt. Trotz unterschiedlicher Darstellungen sollte das Verfahren zu dem Defekt passen und ein geringes Risiko für den Patienten darstellen.

Ein Beispiel für eine lokale Lappenplastik stellt ein zweiseitig gestielter Lappen, erstmalig von Langenbeck 1859 zur Deckung einer Kieferspalte beschrieben, dar [5]. Crawford et al. (1957) beschrieben knapp 100 Jahre später in Bezugnahme auf Stenstrom den Brückenlappen (auch „Visierlappenplastik“), im englischsprachigen Raum „bipedicled/tubed flap“ genannt, in der hier verwendeten Art am Unterschenkel [6, 7]. Heute wird diese Technik durch das Aufkommen und die Verbreitung von freien Lappenplastiken allerdings seltener angewendet oder durch Lappenplastiken wie den Propellerlappen oder einen „keystone flap“ ersetzt. Heutzutage ist die Deckung mit einer Brückenlappenplastik im Kopf-Hals Bereich (beispielsweise Heim et al. (2021)) am weitesten verbreitet [8].

Das Prinzip des Brückenlappens beruht auf einer Parallelverschiebung von unterminiertem und mobilisiertem Gewebe in einen Defekt hinein. Er kann als Nah- oder gestielter Lappen verwendet werden. Aufgrund der sicheren Durchblutung des Lappens („random-pattern flap“) eignet sich der Lappen gut für Defekte in Regionen mit einer eingeschränkten oder vorgeschädigten Durchblutungssituation, wo lokale Keystone oder Propellerlappenplastiken nicht möglich sind und die Gefäßsituation eine freie Lappenplastik risikoreich macht. Weitere Vorteile sind die ähnliche Hautbeschaffenheit von Hebe- und Defektstelle und eine geringe Neigung für eine Gewebeatrophie oder Narbenkontrakturen. Nachteilig ist die Gefahr einer Verziehung von Landmarken und bei Verlust des Lappens ein großer Defekt der Hebestelle [9].

## Methoden

**Abb. 1:**
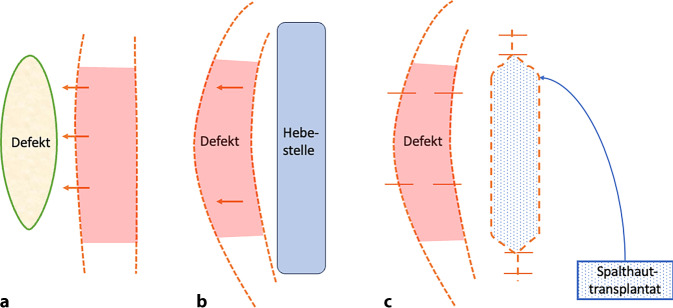
Schematische Darstellung einer Brückenlappenplastik. **a** Es wird eine Inzision (*orange*) mit ausreichender Länge und genügend Basis für den Defekt (*gelb*) durchgeführt. **b** Die Insel (*rot*) wird unterminiert und in den Defekt (*rot*) verschoben. **c** Der Hebedefekt (*blau*) wird anschließend mit Spalthaut (*blau/gepunktet*) gedeckt. Die Brückenlappenplastik (*rot*) deckt den Defekt

Der Lappen wird mit einem Basis-Längen-Verhältnis von 1:2 ausgehend von der Defektgröße geplant. Schwabegger et al. (1966) empfehlen ein Basis-Längen-Verhältnis von maximal 1:4 [10]. Für eine spannungsarme Deckung von breiteren Defekten können zudem 2 Brückenlappen von beiden Seiten gehoben werden [11]. Es erfolgen die konvexe Exzision des Defektes und parallele Inzision zur Hebeseite. Das Gewebe wird subkutan bis in den Defekt unterminiert und mobilisiert (Abb. 1). Anschließend kann der Lappen in den Defekt gehoben und eingenäht werden. Der entstehende Hebedefekt darf dabei keine Sehnen und Knochenteile frei legen [9]. Der entstehende Sekundärdefekt der Hebestelle kann direkt verschlossen, mittels Spalthaut gedeckt oder durch Granulation ausheilen gelassen werden.

Für den hier vorgestellten Defekt über der Fibularisloge wurde die Lappenplastik von medial mit einem Basis-Längen-Verhältnis von 1:2 geplant. Über eine Länge von etwa 20 cm medial der Tibia wurde die Inzision gesetzt, das Hautgewebe epifaszial in den Wundbereich unterminiert und ein 20 cm langer kutaner Lappen in den Defekt nach lateral gehoben. Dabei wurden kranial und kaudal Hautstiele zur Blutversorgung belassen. Die Hebestelle wurde anschließend mit 1:1,5 gemeshter Spalthaut gedeckt.

## Fallbericht

**Abb. 2:**
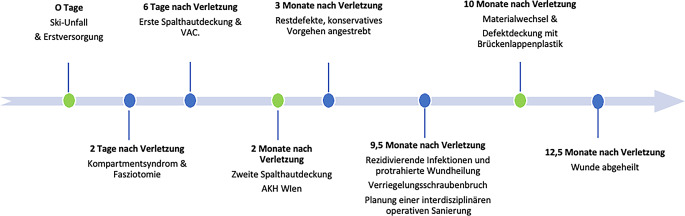
Zeitlicher Verlauf der Krankheitsgeschichte mit sichtbarem prolongiertem Verlauf. *VAC* Vacuum Assisted Closure

Ein 29-jähriger Patient (Abb. 2) zog sich als Folge eines Skiunfalls eine rechtsseitige offene Unterschenkelschaftfraktur am Übergang vom mittleren zum distalen Drittel mit multifragmentärem Keil OTA/AO-Typ 42B3 zu. Die Erstversorgung erfolgte im auswärtigen Spital mittels Verriegelungsnagel (Expert, Fa. DePuy Synthes, Oberdorf, Schweiz) und Ruhigstellung im Gipsverband. Nach 2 Tagen trat ein Kompartmentsyndrom mit der klassischen Symptomatik der „4-P“ („pale, pain, pulseless, paresthesia“) der Fibularis- und Extensorenloge auf. Auf die notfallmäßige Dermatofasziotomie der betroffenen Logen zeigte der Patient eine Besserung der Symptomatik. Ein direkter Verschluss der Fasziotomie war nicht möglich, sodass zur initialen Wundkonditionierung des Defektes eine „Negative Wound Pressure Therapy“/„Vacuum-Assisted-Closure“(VAC)-System-Therapie und anschließend die Spalthautdeckung erfolgte. Zum Zeitpunkt der Deckung bestand allerdings bereits ein massiver Gewebeverlust. Zur weiteren Versorgung wurde der Patient in unser Universitätskrankenhaus transferiert. Hierorts erfolgte eine interdisziplinäre Behandlung des Patienten durch die unfallchirurgische und plastisch-chirurgische Abteilung.

**Abb. 3:**
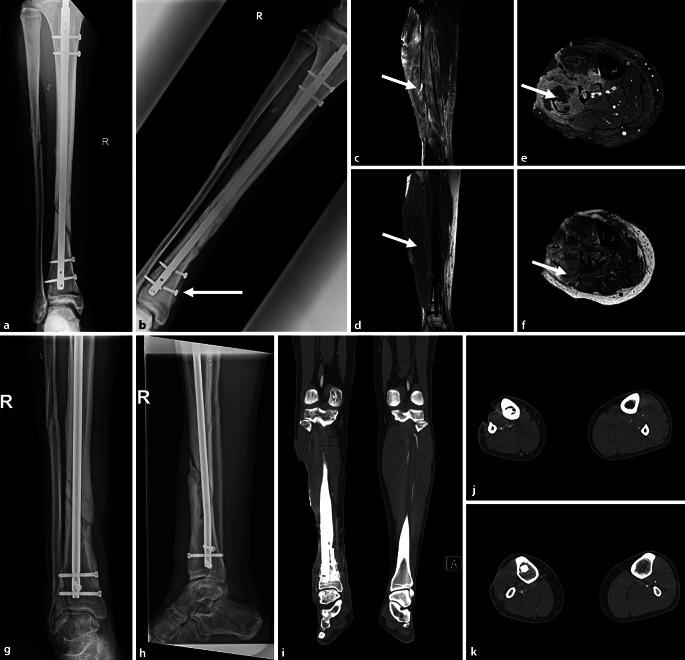
Röntgen-, Computertomograpie(CT)- und Magnetresonanztomographie(MRT)-Bilder des rechten Unterschenkels.** a** Nichtgeheilte Fraktur und Zustand nach Marknagelung 2 Monate nach Trauma, **b** Zustand der Fraktur 8 Monate nach dem Unfall mit gebrochenen Verriegelungsschrauben (*weißer Pfeil*) und der Pseudoarthrose. **c–f** MRT-Untersuchungsergebnisse des akuten Zustands nach Kompartmentsyndrom der Fibularisloge einen Monat nach Trauma mit Weichteildefekt und Atrophie sowie ödematöser Auftreibung der gesamten Flexoren- und Fibularisloge. Die *weißen Pfeile* weisen auf die Fibularisloge mit den Läsionen. **g**, **h** Knöcherne Konsolidierung, mit ventralseitigem Spalt sowie teilweiser Brückenbildung 2 Jahre nach dem Trauma. **i–k** Präoperative lokale Gefäßsituation in der CT-Angiographie ein Jahr nach dem Trauma

Bei Aufnahme zeigte der Patient eine ausgeprägte Schädigung infolge des Kompartmentsyndroms mit einer Läsion des N. peronaeus communis. Die Nervenleitgeschwindigkeitsmessung bestätigte eine hochgradige axonale Läsion ohne jegliche Weiterleitung sensibler und motorischer Signale. Mittels MRT und Biopsien konnte zudem die ausgeprägte Schädigung infolge des Kompartmentsyndroms der Flexor‑, Extensor- und Peronäusmuskulatur bestätigt werden. Dabei zeigten sich die Mm. gastrocnemius, soleus, tibialis anterior sowie die tiefen Flexoren allesamt nekrotisch bzw. fettig degeneriert. Die Gefäßsituation zeigte ebenfalls einen ausgeprägten Befund mit einer abwesenden A. fibularis mit einer konsekutiven Zweigefäßsituation. Die Kollateralgefäße waren in der Angiographie nicht nachweisbar (Abb. 3i–k).

**Abb. 4:**
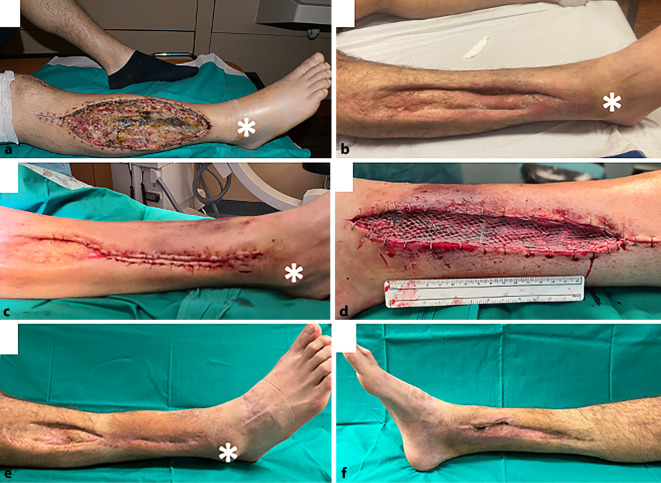
Heilungsverlauf des Patienten. **a** Lokalbefund direkt nach der Übernahme sowie protrahierter Heilungsverlauf 4 Monate nach abermaliger Spalthautdeckung (**b**). **c,** **d** Intraoperativer Zustand nach der Brückenlappenplastik mit der Defektstelle lateral am Unterschenkel (**c**) und der Hebestelle medial (**d**). **e,** **f** Endresultat 9 Monate nach der Brückenlappenplastik mit der gedeckten Defektstelle (**e**) und der Hebestelle (**f**). *Asteriskus* lateraler Malleolus

Das Spalthauttransplantat zeigte im Verlauf nach der Übernahme keine Anheilungstendenz aufgrund eines nekrotischen Untergrunds (Abb. 4a). Es bestand ein Hautdefekt von 10 × 25 cm über der Fibularisloge. Initial wurden mehrmalige Débridements in Kombination mit einer VAC-Konditionierung der Wunde durchgeführt. Nach 6 Wochen erfolgte der zweite Versuch einer Spalthauttransplantation in das bereits stark vorgeschädigte Areal. Bei protrahiertem Heilungsverlauf zeigte sich nach 8 Wochen das vorläufige Zwischenergebnis unter einer größtenteils eingeheilten Spalthaut. Ein 4 × 0,5 cm messender Defekt mit medial frei liegender Tibia blieb bestehen. Dieser wurde vorerst konservativ 6 Monate weiterbehandelt (Abb. 4b). Aufgrund der protrahierten Wundheilung mit offenen Arealen, frei liegender Tibia und erhöhtem Risiko für eine Infektion des Knochens sowie einer ständigen Sezernierung aus dem Hautdefekt wurde ein erneuter Versuch zur Deckung unternommen. Durch die frustrane Gefäßsituation, das stark vorgeschädigte Wundgebiet und den komplikationsbehafteten Verlauf wäre eine Rekonstruktion mittels freier Lappentransplantation mit einer geringen Erfolgswahrscheinlichkeit verbunden. Ebenfalls war der Patient durch den komplexen Verlauf stark vorbelastet und lehnte eine aufwendige Rekonstruktion mittels einer Lappenplastik ab. In der Zusammenschau der lokalen Situation und des bisherigen Verlaufs wurde sich für eine Brückenlappenplastik entschieden.

In einem interdisziplinären Vorgehen erfolgte nach Entfernung des 9 mm im Durchmesser haltenden Tibianagels und der teilweise abgebrochenen Schrauben eine interne Spongiosaplastik durch Aufbohren des Markraums mit anschließender Renagelung auf einen 375 mm langen und 10 mm dicken T2 Tibianagel (Fa. Stryker, Wien, Österreich). Nach Übernahme durch das plastisch chirurgische Team erfolgte der sekundäre Defektverschluss mittels Brückenlappenplastik (Abb. 4c, d). Zwölf Tage postoperativ konnte der Patient in die ambulante Betreuung entlassen werden. In den Kontrollen zeigte sich die Lappenplastik entzündungsfrei eingeheilt. Kleine, zunächst bestehende Restdefekte proximal an der ursprünglichen Wundfläche sowie am Hebedefekt heilten nach 3 Monaten konservativer Therapie vollständig ab. Dadurch konnte der Defekt ein Jahr nach der Verletzung endgültig verschlossen werden (Abb. 4e, f). Die Fraktur ist derweil stabilisiert, befindet sich aber noch in Abheilung (Abb. 4g, h). Mittlerweile hat der Patient wieder mit sportlichen Aktivitäten begonnen. Eine Fallfußsymptomatik durch die Peronäusläsion blieb bestehen und wird mittels Peronäusschiene versorgt.

## Diskussion

Die anatomischen Begebenheiten mit einem besonders dünnen Weichteilmantel über der Tibia führen bei traumatischen Verletzungen des Unterschenkels häufig zu frei liegenden Knochenanteilen. Über diesen Defekten ist die Prognose für eine Defektdeckung mit Spalthaut schlecht, wobei gleichzeitig die Knochenheilung stark beeinträchtigt ist. Die Schwierigkeit bei der Deckung stellt die ausreichende Gefäßversorgung dar. Vogt et al. (2011) nennen als typische Indikation für den Brückenlappen eingeschränkte Durchblutungsverhältnisse insbesondere am Unterschenkel. Der Lappen zeigt dabei eine hohe Erfolgsrate durch die gute Durchblutung, bei naturgemäß eingeschränkter Bewegungsfreiheit [9]. Bei unserem Patienten lag eine stark eingeschränkte Durchblutungssituation durch das ausgeprägte Kompartmentsyndrom der Fibularisloge statt. Die A. fibularis ab der Abzweigung sowie die Gefäßäste in der Fibularisloge waren nicht mehr vorhanden. Es bestand eine Trümmerzone distal des Kniegelenks. Zudem zeigte sich das umliegende Gewebe stark fibrotisiert und narbig verändert. Die nächsten zielführenden Anschlussgefäße würden sich erst proximal vom Kniegelenk befinden. Schwabegger et al. (1996) beobachteten darüber hinaus gute Erfolge der Brückenlappenplastik bei Defekten an der unteren Extremität mit frei liegenden Knochen oder Sehnenstrukturen in einer Situation, in der eine Hauttransplantation nicht möglich ist [10]. Granzow et al. (2013) zeigten bei 10 Patienten ebenso zufriedenstellende Ergebnisse mit einem Brückenlappen am Unterschenkel und nannten die Technik eine verlässliche und einfachere Alternative zu freien Lappen [11]. Dabei sollte die Planung entsprechend der lokalen Situation erfolgen. Bei einem stark ödematösen Befund muss zuvor die Möglichkeit eines Verschlusses überprüft und ggf. eine Abschwellung abgewartet werden. Es kann bei einem großen Defekt, auf eine doppelseitige Brückenlappenplastik ausgeweitet werden [11]. In der Arbeit von Pollak et al. (2010) wurde gezeigt, dass freie Lappen zur Defektrekonstruktion an der unteren Extremität bei Typ-C-Frakturen nach Einteilung der Arbeitsgemeinschaft für Osteosynthesefragen generell weniger Komplikationen bei der Wundheilung aufweisen als lokale Lappenplastiken. Für niedergradige Verletzungen, wie im vorliegenden Fall, wurde allerdings kein signifikanter Vorteil von freien Lappen festgestellt [12].

In Übereinstimmung mit der genannten Literatur erschien uns die Deckung mittels Brückenlappen als einzige zielführende und sichere Methode zur Rekonstruktion. Eine freie Lappenplastik wäre als weitere Eskalation anschließend immer noch durchführbar gewesen, allerdings aufgrund der fragilen Gefäßsituation unter erschwerenden Voraussetzungen mit einer höheren erwartbaren Komplikationsrate.

## Fazit für die Praxis

Die Brückenlappenplastik erwies sich im vorgestellten Fall als verlässliche Alternative zu einer freien Lappenplastik, insbesondere bei schwieriger Gefäßsituation. Die Technik bietet sich besonders bei länglichen Defekten mit frei liegenden Sehnen- oder Knochenanteilen an, wo eine Lappendeckung nötig ist, bei gleichzeitig schlechten Gefäß- bzw. Durchblutungsverhältnissen. Als Möglichkeit für eine stark vorgeschädigte Umgebung mit einem komplikationsbehafteten Wundverlauf konnte mit der Brückenlappenplastik nach über einem Jahr ein endgültiger und stabiler Wundverschluss erzielt werden. In der Zusammenschau stellt die Brückenlappenplastik nach wie vor eine sichere Rekonstruktion selbst von sehr komplexen Wunden dar und sollte stets als Alternative zu freien Lappenplastiken in die Therapieentscheidungen genommen werden.
